# Role of N6-Methyladenosine (m6A) epitranscriptomic mark in regulating viral infections and target for antiviral development

**DOI:** 10.3389/fphar.2025.1667283

**Published:** 2025-09-12

**Authors:** Mahmoud Bayoumi, Vidya Manju, Luis Martinez-Sobrido, Muhammad Munir

**Affiliations:** ^1^ Host-pathogen interactions (HPI) and Disease Intervention and Prevention (DIP) programs, Texas Biomedical Research Institute, San Antonio, TX, United States; ^2^ Virology Department, Faculty of Veterinary Medicine, Cairo University, Giza, Egypt; ^3^ Division of Biomedical and Life Sciences, Faculty of Health and Medicine, Lancaster University, Lancaster, United Kingdom

**Keywords:** antiviral, drug, m6A, RNA, therapeutics, viral infection

## Abstract

Viral infectious diseases continue to pose significant public health threats, driving severe epidemics and occasional pandemics of great consequences to humans. Viral infections trigger a range of transcriptional and epitranscriptional changes, including N6-methyladenosine (m6A) modification—one of the most abundant and dynamic RNA methylation marks. Although m6A mark was identified decades ago, its functional relevance in viral RNA remained elusive until recent advances in sequencing technologies. Viruses, like their host cells, depend on mRNA for protein synthesis and must rapidly replicate and evade host immune responses. This review focuses on the critical role of m6A in the regulation of viral infections and immune responses. Herein, we explore the most recent advances on how viruses exploit the m6A marks and host m6A machinery to enhance their replication and how host m6A modifications can influence viral pathogenicity. Understanding the interplay between m6A modifications and viral life cycles will be important for the potential of targeting m6A regulatory proteins as novel antiviral strategies to control viral infections. Moreover, a better understanding of these mechanisms will contribute to deeper insights into the host innate immune response and the development of innovative antiviral therapeutics.

## 1 Introduction

As early as the 1960s, the non-canonical nucleotides were noticed with the emergence of the nucleotide sequencing era. Other than A, G, U, and C, the pseudouridine (Ψ) was reported in the first RNA sequencing of the tRNA isolated from yeast, frequently named the fifth nucleotide (Cohn, 1960; Holley et al., 1965). Later, it was identified that the long non-coding RNA species (lncRNA, including tRNA, rRNA, and spliceosomal RNA) carry a massive diversity of modified nucleosides with crucial biological functions. Transfer RNA (tRNA) has been noted to contain many modified bases compared to other RNA species found in eukaryotic cells. On average, a single tRNA molecule possesses 13 modifications, including methylation of ribose sugars and nucleobase and base isomerization. These modifications play a crucial role in ensuring the correct folding and stability of tRNA molecules, thereby enhancing decoding fidelity to its highest extent (Roundtree et al., 2017). In a similar manner, ribosomal RNA (rRNA) displayed a minimum of 200 modifications necessary for accurate translation in eukaryotic organisms. It is important to highlight that the removal of pseudouridine or methylated ribose inhibits the biogenesis of rRNA. Comparable findings were also observed regarding RNA modifications in spliceosomal RNA (Roundtree et al., 2017).

The well-established modifications that occur post-transcriptionally on pre-mRNA include 5′capping and the addition of a poly(A) tail. These known modifications play crucial roles in ensuring transcript stability and initiating translation in eukaryotic cells. Notably, the discovery of methylation at the 5′cap of mRNA has led several research groups serendipitously also to identify the methylation of internal bases (Desrosiers et al., 1974; Perry and Kelley, 1974; Adams and Cory, 1975). It has been proposed that these modifications may have functional regulatory roles, similar to the methylation marks found on cellular histones and DNA; however, the functional role for different aspects of biology was not identified until 2012 with the advent of m6A-seq analysis (Dominissini et al., 2012; Meyer et al., 2012). The most frequently observed methylation of adenosines includes N6-methyladenosine (m6A), N1-methyladenosine (m1A), N6,2′-O-dimethyladenosine (m6A.m.), methylation of the ribose sugar in specific bases (Nm), and 5-methylcytidine (m5C). Collectively, these chemical modifications in the mRNA are referred to as the epitranscriptome (Roundtree et al., 2017). This review specifically focuses on the predominant methylation mark, m6A, particularly in relation to viral infections and immune regulatory aspects, highlighting that understanding the role of m6A in these contexts could pave the way for new antiviral therapies.

## 2 The epitranscriptomic timeline of m6A marks on mRNA

In the 1970s, m6A marks were detected in hepatoma cells (Desrosiers et al., 1974). After that, these marks were recorded in various organisms, including bacteria (Deng et al., 2015), yeast (Agarwala et al., 2012), plants (Yue et al., 2019), mice (Dominissini et al., 2012), and humans (Meyer et al., 2012). The m6A has been reported to control various RNA metabolic functions, including translation, splicing, secondary structure, and stability (Li and Mason, 2014; Meyer and Jeffery, 2014). Moreover, m6A signatures are involved in various biological functions, including embryogenesis, mice fertility, and cellular differentiation, suggesting essential regulatory roles in cellular lifecycles (Niu et al., 2013; Zheng et al., 2013).

As mentioned earlier, the methylation of adenosine was noticed nearly 5 decades ago. However, the methods utilized at this time were labelling cellular RNA followed by thin-layer chromatography techniques. These techniques usually provide an idea about the relative abundance of methylated residues. It has been reported that the m6A marks are located every 0.7–0.8 kb in the mRNA and 2–3 kb in the lncRNA (Lavi et al., 1977). Additionally, the labelling techniques followed by nucleic acid digestion displayed that the m6A marks were enriched predominantly in consensus sequence, the GA*C > AA*C sequences (here A* denotes the methylatable adenosine) (Wei and Moss, 1977). Recently, we and others confirmed this signature and found to be conserved among various hosts and viruses (Dominissini et al., 2012; Linder et al., 2015; Bayoumi and Munir, 2021a).

However, the scientific community was reluctant to accept the m6A marks as biologically crucial in eukaryotes until recently. Two significant breakthroughs occurred to get the m6A marks back on track. Firstly, Jia et al. (2011) identified the first m6A demethylase enzyme. This finding indicates that the installation of the m6A marks has biological regulatory roles and is a reversibly dynamic process (Jia et al., 2011). In follow-up research, the same group identified the second m6A demethylase, ALKBH5, supporting the critical regulatory functions in eukaryotic cells, including proper metabolism and spermatogenesis (Zheng et al., 2013).

Secondly, at the same time, two independent groups developed a new high-throughput sequencing method for the methylated RNA (m6A-seq or MeRIP-seq) to relatively identify the m6A topology in human mRNAs in different tissues in a transcriptome-wide approach (Dominissini et al., 2012; Meyer et al., 2012). Through these methods, the location and function of m6A in a given mRNA could be determined. Notably, the m6A-seq identified m6A marks in a 100–200 nucleotide window. It has been reported that the m6A methylome is relatively conserved between humans and mice (Dominissini et al., 2012). This finding also supports that the m6A marks have evolutionarily conserved functions among species.

Notably, in contrast to m6A, the incorporation of m1A interferes with the Watson-Crick base-pairing model, resulting in a significant structural change in the RNA secondary structure and its interaction with proteins. m1A is found in lower quantities in mRNA transcripts compared to m6A and can be removed by the enzyme ALKBH3. The role of m1A is believed to enhance protein translation efficiency (Dominissini et al., 2016). Furthermore, m6A.m., which is predominantly located at the first nucleotide following the m7G cap in mRNA, has also been identified in the eukaryotic methylome, where it contributes to RNA stability and protects against mRNA degradation (Mauer et al., 2017). Nevertheless, further research is required to fully elucidate the functions of these modifications in various biological processes, which is beyond the scope of this review.

## 3 m6A-associated machinery regulating viral infection

As previously mentioned, m6A marks have gained significant recognition for their role in regulating cellular functions due to their dynamic regulatory processes. A complex of proteins is involved in the deposition of m6A on the candidate pre-mRNA. This complex includes an active component known as methyltransferase-like-3 (METTL3), which is structurally supported by the METTL14 protein (Wang X. et al., 2016; Wang et al., 2016 P.). Furthermore, the Wilms tumour 1-associated protein (WTAP) plays a crucial role in directing the entire complex to nuclear speckles, thereby enhancing methylation efficiency (Ping et al., 2014). It is important to note that a variety of cofactors, such as KIAA1429, RBM15, HAKAI, and ZC3H13, regulate m6A methylation. The structure and function of m6A writers have been addressed in other studies (Huang and Yin, 2018).

As a cellular dynamic process, the m6A marks are reversed using one of the two well-identified enzymes to demethylate mRNA. The FTO and ALKBH5 belong to the Alkb-homolgue family members to passively demethylate m6A-containing mRNA into adenosine. However, both differ in tissue distribution; FTO is mainly enriched in brain tissues, whereas ALKBH5 is predominantly enriched in the testes. Furthermore, ALKBH5 is expressed primarily in the nucleus, while FTO is expressed in both the nucleus and cytoplasm (Jia et al., 2011; Zheng et al., 2013; Meyer and Jaffrey, 2017). Additionally, both differ greatly in their substrate specificity. The ALKBH5 demethylates only the methylated adenosines. FTO utilizes 3mU, m6A, m1A, and m6A.m. in various RNA species, as we reviewed earlier (Bayoumi and Munir, 2021c). However, recent reports showed that FTO predominantly demethylates the m6A.m. (Verhamme et al., 2025).

**FIGURE 1:**
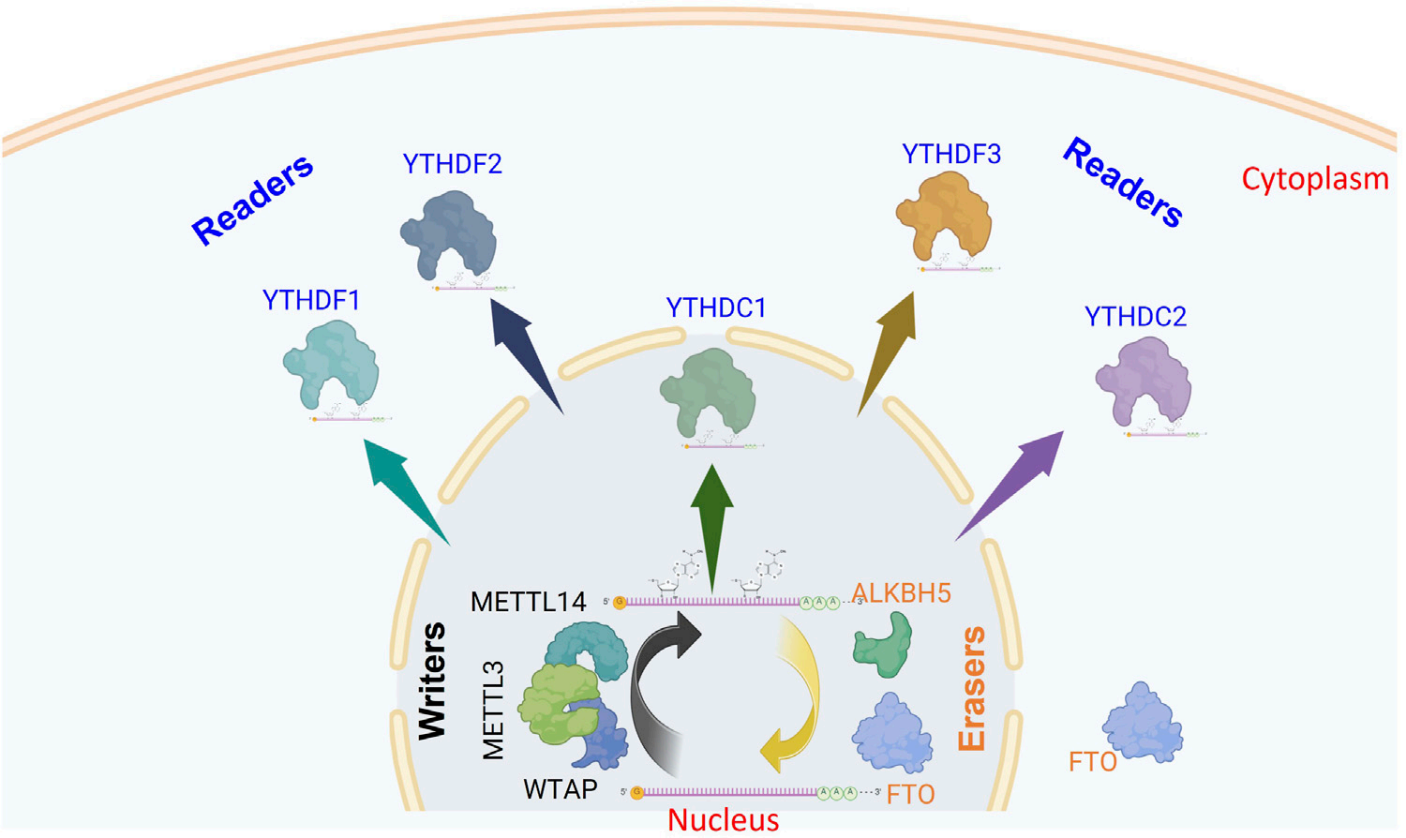
Schematic representation of m6A modification and its regulatory machinery. This diagram illustrates the process of m6A modification in mRNA and the core components involved. In the nucleus, m6A marks are added to pre-mRNA by a complex of methyltransferase enzymes (writers). These modifications can be removed by demethylases (erasers), which function independently. Once methylation occurs, m6A reader proteins recognize and bind to these marks, triggering downstream biological effects either in the nucleus (YTHDC1) or the cytoplasm (YTHDF1–3, and YTHDC2). This illustration highlights the ten principal proteins constituting the m6A regulatory machinery (The figure was created using BioRender).

The m6A methylated RNA binds to various RNA-binding proteins; the most important are YTH-domain-containing proteins in the nucleus, YTHDC1, or cytoplasm, YTHDF1-3 and YTHDC2 (readers). The interacting reader protein exerts a specific function on the methylated transcripts that dictates the fate of RNA and cell biology. The nuclear YTHDC1 predominantly induces exon inclusion to mRNAs through recruitment of certain splicing factors (Xiao et al., 2016). Whereas YTHDF1 promotes translation by enhancing ribosome loading and binding to initiation factors (Wang et al., 2015). In contrast, YTHDF2 regulates RNA metabolism via decreasing RNA stability and promoting RNA decay (Wang et al., 2014; Du et al., 2016). Interestingly, YTHDF3 demonstrated synergistic roles with YTHDF2 to promote RNA decay or interact with YTHDF1 to enhance protein translation, suggesting the cooperative manner of the cytoplasmic YTHDF1-3 proteins to impact the biological processes (Shi et al., 2017). YTHDC2 was reported to improve translation efficiency and promote normal spermatogenesis in mice (Hsu et al., 2017). These components are known as the m6A machinery (Figure 1).

## 4 Role of epitranscriptomic modifications in regulating viral infection

Viruses rely on mRNA to produce their proteins, utilizing the host’s cellular machinery to facilitate replication. However, they face constant pressure to rapidly synthesize RNA, express proteins, and replicate in order to evade the immune response and gain an advantage in the ongoing virus-host battle. Recently, the association between epigenetic and epitranscriptomic control and the establishment of viral infection has begun to arise. Generally, eukaryotic cells can exploit the epigenetic forces as an antiviral response against a wide range of viruses. In turn, DNA viruses exploit cellular epigenetic silencing mechanisms to establish a latent infection cycle (Knipe et al., 2017). Interestingly, viral RNA accepts this m6A decoration as well, suggesting that the viral RNA uses the epitranscriptomic marks to dictate the viral lifecycle (Kennedy et al., 2017; Baquero-Perez et al., 2021).

As indicated earlier, viruses are under continued pressure to replicate rapidly. One of the mechanisms by which viruses can enhance replication and protein expression is through acquiring/losing chemical modifications compared to their cellular mRNA counterparts. Scanty chemical modifications are currently known to regulate viral replication and gene expression, including the m6A, 5-methylcytidine (m^5^C), N4-acetylcytidine (ac^4^C), and 2′O-methylation of the ribose moiety of the ribonucleosides (referred to as Nm). Fascinatingly, viruses accommodate 2-10 times m6A and m5C marks higher than their cellular counterparts. Similarly, the Nm is 10–30 times higher than cellular RNA (Courtney et al., 2017; 2019b; 2019a). All these increased levels of modified transcripts enhance viral genome replication and gene expression through either enhanced mRNA stability (m6A, ac4C), mRNA translation (m6A, m5C), or evasion of immune responses (m6A, Nm). However, these previous findings only represent influenza A virus (IAV) and retroviral models (HIV-1, and MLV). Therefore, investigating more viruses would support the conclusion that RNA modifications are associated with the replication of more viruses. In contrast, other virus models oppose this hypothesis; various flaviviruses, including HCV and Zika virus, have been reported to have reduced virus replication with more m6A levels (Gokhale et al., 2016; Lichinchi et al., 2016b). It is arguably why highly evolving viruses could keep an evolutionary mark if it is indeed inhibitory. These discrepancies warrant more investigations in m6A virus-related fields.

## 5 The interplay between the m6A modification and viral infection

Several decades ago, m6A marks were identified to be incorporated in viral RNAs. However, due to technological limitations, the topological and functional characteristics of epitranscriptomic m6A marks were not clearly defined in viral-host interaction (Lavi and Shatkin, 1975; Hashimoto and Green, 1976; Krug et al., 1976; Kane and Beemon, 1985; Narayan et al., 1987). In recent years, progress in epitranscriptome-wide sequencing technologies has been exploited to identify and relatively quantify m6A marks (Hafner et al., 2010; Meyer et al., 2012; Liu et al., 2013; Chen et al., 2015; Linder et al., 2015; Price et al., 2020). These technologies have been harnessed, unravelling aspects of the m6A marks in understanding host-pathogen interactions, as shown below. The outcomes are described in relation to the Baltimore system of virus classification, as follows:

### 5.1 Class I viruses: double-stranded DNA viruses

#### 5.1.1 The role of m6A in regulating viruses belonging to the *Herpesviridae* family

##### 5.1.1.1 Herpes virus type 1 (HSV-1)

Unlike most RNA viruses, DNA viruses have access to most m6A machinery. Herpesviruses have been reported to carry m6A marks since the 1970s (Moss et al., 1977). A comprehensive study has established that m6A plays a positive role in the lifecycle of HSV-1. Introducing the chemical 3-deazaadenosine (DAA) diminishes the availability of the SAM methyl donor, reducing cellular m6A mark deposition and resulting in a more than 1000-fold decrease in viral replication (Feng et al., 2021). In contrast, the overexpression of METTL3 promotes viral replication, while its knockdown reduces this process. A similar inverse effect was observed when cells were treated with m6A-erasers. Notably, the depletion of YTHDF3 resulted in a significant 90% decrease in viral replication. These results clearly indicate that m6A positively influences HSV-1 replication, suggesting that targeting m6A machinery may serve as an effective antiviral approach. Nevertheless, the underlying mechanisms of these observations remain to be explored (Feng et al., 2021).

A recent study has confirmed that HSV-1 reprograms the m6A machinery to enhance the oncolytic properties of HSV-1 (oHSV-1). The mechanism involves the viral ICP0 protein, which acts as a ubiquitin E3 ligase, leading to the ubiquitination and degradation of METTL14. This process results in the downregulation of the interferon-stimulating gene 15 (ISG15), thereby inhibiting antiviral responses and promoting viral replication. Furthermore, the silencing of METTL14 significantly increased the anti-tumor effects induced by oHSV-1, indicating that the METTL14/ISG15 pathway may serve as a promising therapeutic target for HSV-1 infections. Additionally, a METTL14-specific inhibitor could potentially enhance the efficacy of oHSV-1 in clinical settings, offering renewed hope for oncolytic herpes virotherapy (Chen Y. et al., 2024).

##### 5.1.1.2 Human cytomegalovirus (HCMV)

The m6A machinery has been proposed to play a pivotal role in HCMV through a negative interferon (IFN) response mechanism. A significant reduction in HCMV titre was observed in m6A writer and reader knockout cells (Winkler et al., 2019). Interestingly, interferon β (IFNβ) mRNA was found to be m6A modified in METTL3-and YTHDF2-depleted cells and was highly stabilized. The same results were found upon introducing the UV-treated virus, suggesting a non-viral mechanism controlling the replication in knockout cells. Mechanistically, the m6A modifications are negative regulators of IFNs by controlling the fast turnover of IFN mRNAs and thus enhancing viral proliferation (Winkler et al., 2019).

##### 5.1.1.3 Kaposi’s sarcoma-associated herpesvirus (KSHV)

Like other herpesviruses, KSHV mRNA undergoes m6A modifications, and m6A-modified mRNAs increased markedly during KSHV lytic replication. Moreover, inhibition of m6A marks on replication transcription activator (RTA; an essential switch protein during the transition to lytic infection) halts the KSHV lytic cycle (Ye et al., 2017). Additionally, FTO knockdown increased m6A levels and enhanced lytic gene expression, whereas knockdown of METTL3 had the opposite effects. This information indicated a proviral impact of m6A in the KSHV lytic cycle (Ye et al., 2017). In primary effusion lymphoma (BCBL-1) cells, others noted that YTHDFs protein members had a positive role in the viral lytic cycle; more interestingly, authors identified the staphylococcal nuclease domain-containing protein 1 (SND1), as a novel m6A-reader in the KSHV lytic cycle. Structural analysis showed that SND1 has an aromatic cage similar to the YTH domain identified in the YTHDFs and has a proviral effect in the KSHV lytic cycle (Baquero-Perez et al., 2019).

It has also been reported that the knockdown of YTHDF2 and METTL3 in renal carcinoma (iSLK) cells predominantly reduces viral gene expression and virion production. Intriguingly, the same report also showed that YTHDF2 and METTL3 depletion have the opposite effect on viral gene expression in TREx BCBL-1 cells, suggesting that m6A has a central role in regulating KSHV and functioned in a pro- and antiviral manner according to the investigated cell lines (Hesser et al., 2018). Adding more layers of complexity in understanding the effect of m6A machinery in regulating KSHV, another investigation revealed that the knockdown of YTHDF2 increased viral gene expression. Mechanistically, YTHDF2 facilitates viral transcript degradation, thus inhibiting the KSHV lytic life cycle in iSLK cells (Tan et al., 2018). Overall, all these studies revealed that m6A modifications play critical roles in the KSHV life cycle; however, the functional role of YTHDF2 remains unclear, and the discrepancy needs to be fully addressed.

##### 5.1.1.4 Epstein - Barr virus (EBV)

The association between the functional role of m6A installed onto EBV transcripts and EBV lytic and latent cycles, and EBV-associated cancers, was also elucidated (Lang et al., 2019). It has been reported that METTL14 was markedly increased during EBV latency and reduced during lytic infection. The study also demonstrates that Epstein-Barr nuclear antigen 3C (EBNA3C), a viral-encoded oncoprotein involved in interaction and activation of METTL14, promotes ts stability. In this way, EBNA3C exploits METTL14 to regulate tumour formation (Lang et al., 2019). It has also been confirmed that YTHDF1 plays a crucial role in reducing the replication of EBV. YTHDF1 achieves this by destabilizing primary viral transcripts such as BZLF1 and BRLF1 through the recruitment of destabilizing factors, indicating its function as an antiviral agent in the regulation of EBV (Xia et al., 2021).

Recent research has shown that EBV infection reduces the levels of m6A modifications on toll-like receptor (TLR)9, consequently inhibiting its expression (Zhang et al., 2024). Moreover, the silencing of METTL3 or using METTL3 inhibitors decreased TLR9 protein levels due to reduced mRNA stability. Mechanistically, Epstein-Barr nuclear antigen 1 (EBNA1) promotes the degradation of METTL3 protein through K48-linked ubiquitination. Furthermore, YTHDF1 enhances TLR9 expression by facilitating mRNA translation in an m6A-dependent manner. This indicates that EBV may impede TLR9 translation by manipulating the host’s m6A modification processes. This study uncovers a novel mechanism by which EBV suppresses the crucial innate immunity molecule TLR9 by modulating the host’s m6A modification system (Zhang et al., 2024).

##### 5.1.1.5 Alphaherpesvirus pseudorabies virus (PRV)

PRV is another salient model for reprogramming m6A marks and m6A machinery to regulate viral infection. Verhamme et al. (2025) demonstrated that the UL13 protein kinase of PRV initiates post-translational phosphorylation of FTO, the m6A and m6A.m. erasers. Viral UL13 facilitates FTO-dependent inhibition of ISGs expression. In primary epithelial cells, the reduction of FTO results in an elevated expression of antiviral ISGs (Verhamme et al., 2025), suggesting that FTO inhibitors may show yet-to-be-determined strategies to modulate the antiviral IFN response to regulate viral infection (Verhamme and Favoreel W., 2025).

#### 5.1.2 The role of m6A in regulating viruses belonging to the *Adenoviridae* family

The first documentation of m6A-modified adenoviruses dates back to the 1970s (Hashimoto and Green, 1976). Due to the intricate nature of the adenovirus genome and transcriptome, a combination of m6A sequencing and direct RNA long-read nanopore sequencing was utilized. This research confirmed that adenovirus serotype 5 (Ad5) undergoes m6A modification. The findings indicated that METTL3 plays a positive role in regulating the replication of Ad5, while the other components of the m6A machinery did not influence viral replication. The study revealed that the absence of METTL3 specifically affects late viral mRNAs by diminishing their splicing efficiency (Price et al., 2020).

#### 5.1.3 The role of m6A in regulating viruses belonging to the *polyomaviridae* family

Since the 1970s, m6A modifications have been detected in transcripts of Simian Virus 40 (SV40), a member of the *Polyomaviridae* family known for its association with tumor development (Lavi and Shatkin, 1975; Canaani et al., 1979). However, the precise functional significance of these m6 modifications remained unclear until recent advancements in high-throughput m6A sequencing technologies. Tsai et al. (2018) identified 13 m6A sites within SV40 transcripts, with two sites being detected early in the viral lifecycle and 11 identified in late viral transcripts (Tsai et al., 2018). The study also found that YTHDF2 and METTL3 play a crucial role in enhancing viral genome replication and gene expression. Conversely, loss-of-function experiments targeting YTHDF2 and METTL3 resulted in reduced viral activity. Moreover, introducing synonymous mutations that disrupted the mapped m6A sites in late viral transcripts led to a decrease in viral gene expression, indicating that m6A serves as a positive regulator of SV40 (Tsai et al., 2018).

### 5.2 Class II viruses: single-stranded DNA viruses

Although this class contains many viruses of significant importance, no data on the impact of m6A on their replication have been published so far.

### 5.3 Class III viruses: double-stranded RNA viruses

#### 5.3.1 The role of m6A in regulating viruses belonging to the *Reoviridae* family

##### 5.3.1.1 Rotavirus (RV)

A recent report showed that RV infection substantially increased cellular m6A methylome and selectively downregulated ALKBH5. Through m6A-seq analysis, it has been noticed that the IFN regulatory factors 7 (IRF7) carry enrichment of m6A and thus modulates viral infection, possibly through stable and sustained expression. Moreover, METTL3-depleted mice showed an enhanced immune response to ensure rapid virus clearance through IRF7 upregulated pathway in an m6A-dependent manner. Interestingly, RV restored its antiviral activity after depleting IRF7 in METTL3-deficient mice (Wang et al., 2022). This report highlights the significance of m6A in the regulation of the dsRNA viral model and indicates that the depletion of a crucial component of the m6A modulatory protein correlates with an improved immune response in a manner dependent on m6A, whether directly or indirectly.

### 5.4 Class IV viruses: single-stranded RNA, positive sense viruses

#### 5.4.1 The role of m6A in regulating viruses belonging to the *Picornaviridae* family

##### 5.4.1.1 Enterovirus-71 (EV71)

Hao et al. (2019) reported that RNA undergoes m6A modifications using MeRIP-seq analysis and showed that m6A sites are primarily enriched at viral structural proteins (VPs), including VP1 and VP3. Almost all components of the m6A cellular machinery were affected by EV71 infection, and almost all the nuclear m6A machinery translocated to the cytoplasm upon stimulation with this cytoplasmic-replicating virus (Hao et al., 2019). Moreover, METTL3/14 and YTHDF proteins played a proviral role in regulating EV71 in Vero cells, while FTO had a negative regulatory role. It was also observed that ALKBH5 fails to modulate the EV71 life cycle. A marked reduction in viral replication was also noticed when *bona fide* selected m6A sites located on viral genomes were ablated. Therefore, the m6A residues in EV71 mRNA played a positive role in viral replication (Hao et al., 2019). Interestingly, the same report confirmed that YTHDF proteins had an antiviral role in the RD cell line (Hao et al., 2019). In follow-up mechanistic studies, the role of METTL3 in modulating antiviral responses to promote EV71 replication was elucidated (Hao et al., 2024). The authors discovered that METTL3 mediates an m6A-dependent inhibition of the antiviral response by concealing viral RNA from recognition by RIG-I sensors. They also uncovered a non-m6A-dependent mechanism through which METTL3 stabilizes DEAD-box helicase 3X (DDX3X), thereby preventing its degradation and facilitating EV71 infection (Hao et al., 2024).

##### 5.4.1.2 Coxsackievirus B3 (CVB3)

The genome of CVB3 has been found to bear m6A modifications, and it has been noted that CVB3 infection can alter the expression and distribution of m6A-related components within infected cells. The authors reported that 3-deazaadenosine (3-DAA), an m6A modification inhibitor, significantly impairs CVB3 replication. Additionally, METTL3 and METTL14 are shown to enhance CVB3 replication, whereas the m6A demethylases FTO and ALKBH5 have opposing effects. Reducing the levels of m6A-binding proteins such as YTHDF1, YTHDF2, and YTHDF3 led to a significant decline in CVB3 replication, indicating their role in positively regulating CVB3 replication through the modulation of YTHDF-mediated stress granule dynamics (Zhao H. et al., 2024; Zhao et al., 2024 G.). This report also suggests a potential therapeutic approach for CVB3-induced myocarditis by targeting m6A and its associated machinery. A similar phenotype was also observed in CVB1 within human pancreatic beta cells (Bonfim et al., 2024).

##### 5.4.1.3 Foot-and-mouth disease virus (FMDV)

In a recent study, authors discovered that the host protein GTP-binding protein 4 (GTPBP4), a multifunctional cellular protein, acts as a negative feedback regulator of innate immune responses. Knocking out GTPBP4 enhances the antiviral innate immune response *in vitro*, thereby inhibiting replication of FMDV. Moreover, mice lacking GTPBP4 exhibit increased resistance to FMDV infection. To counteract the host’s antiviral immunity, the structural protein VP1 of FMDV elevates the expression of GTPBP4. Mechanistically, FMDV VP1 induces autophagy during viral infection and interacts with m6A reader YTHDF2, leading to its degradation via an AKT-MTOR-dependent pathway, elevated GTPBP4 mRNA and protein levels. The increased GTPBP4 subsequently inhibits the binding of IRF3 to the IFNβ promoter, thereby suppressing the production of type I IFN during FMDV infection (Liu H. et al., 2024).

#### 5.4.2 The role of m6A in regulating viruses belonging to the *Flaviviridae* family

Gokhale et al. (2016) have also demonstrated that most members in the *Flaviviridae* family, including hepatitis C, Zika, yellow fever, West Nile, and dengue viruses, were edited by m6A marks, and these were relatively conserved in the family. Intriguingly, they reported that m6A had a negative impact on hepatitis C virus (HCV) production. Knockdown of m6A methyltransferases increased virion production, while FTO, but not ALKBH5, had the opposite effect. Additionally, they reported the colocalization of YTHDFs with lipid droplets to regulate virion release negatively, indicating that m6A had a negative regulatory effect on the HCV lifecycle. To demonstrate the functional relevance of m6A directly impacting the HCV lifecycle, m6A-abrogating mutations in the virion genome increased virus production (Gokhale et al., 2016). Another independent study confirmed that Zika virus (ZIKV) RNA is m6A modified and supported the negative regulatory role of YTHDFs and methyltransferases on virus replication and protein expression (Lichinchi et al., 2016b). The rationale behind highly evolving viruses in maintaining the epitranscriptomic marks, if they are indeed inhibitory, needs further explanation.

It has also been reported that stimulation of various members of the *Flaviviridae* family significantly increased cellular m6A methylome in an m6A-dependent manner. Some of the stimulated transcripts control *Flaviviridae* infection accordingly, either by regulating protein expression (i.e., RIOK3) or splicing (i.e., CIRBP) (Gokhale et al., 2020). Additionally, m6A modification of HCV pathogen-associated molecular patterns (PAMPs) was reported to reduce recognition by retinoic acid-inducible gene-I (RIG-I), and YTHDFs protect methylated transcripts from cell innate immune sensing (Kim G. W. et al., 2020). Overall, m6A controls the *Flaviviridae* infection cycle and the cellular methylome against innate immune response.

On the other side, METTL14 has a proviral effect on classical swine fever virus (CSFV). Mechanistically, CSFV NS5B protein played a crucial role in taking control of HRD1 and preventing the ubiquitination modification of METTL14. Subsequently, CSFV facilitates m6A modification of TLR4 mRNA through METTL14, while YTHDF2 identifies and promotes the degradation of the modified TLR4 mRNA. This process reduces TLR4 protein levels and subsequently inhibits the NF-κB pathway, thereby enhancing CSFV replication (Chen J. et al., 2024).

#### 5.4.3 The role of m6A in regulating viruses belonging to the *Togaviridae* family

##### 5.4.3.1 Chikungunya virus (CHIKV)

In an elegant study, a 4-thiouracil (4sU)-labeled CHIKV was used to infect cells and pre-replicated viral genome and interacting cellular proteins were identified by mass spectrometry. CHIKV was determined to harbour m6A marks, and YTHDF1 was among the interacting RNA-binding proteins (RBPs) that significantly downregulated virus replication. Investigating YTHDFs revealed various outcomes for CHIKV infection, where YTHDF-1 and -3 restricted virus replication, and YTHDF2 promoted it. Other m6A machinery and the mechanistic effect of YTHDFs in regulating CHIKV infection warrant further investigations (Kim B. et al., 2020).

It is noteworthy to mention that a recent report showed that the m6A marks are not a general trait for cytoplasmic replicating viruses (Baquero-Pérez et al., 2024), in contrast to earlier studies that highlighted the role of m6A in regulating DENV and CHIKV (Baquero-Pérez et al., 2024) argue that these viruses do not possess m6A methylation. They attribute the discrepancies to the reliance on m6A-seq analysis, which is an antibody-dependent method. By combining m6A-seq with non-antibody-dependent techniques such as the novel SELECT and nanopore direct RNA sequencing, they confirmed the absence of m6A methylated transcripts in these cytoplasmic viruses (Baquero-Pérez et al., 2024). This data suggests the need for an orthogonal sequencing approach to validate the findings from m6A sequencing data. These findings underscore the importance of methodological rigor and cross-validation in epitranscriptomic research and highlight the need for standardized approaches to accurately define the role of m6A in viral infections.

#### 5.4.4 The role of m6A in regulating viruses belonging to the *Coronaviridae* family

##### 5.4.4.1 Porcine epidemic diarrhoea virus (PEDV)

The m6A marks are readily expressed in PEDV, which infects mainly young piglets (Chen et al., 2020). Interestingly, m6A-seq analysis indicated seven peaks located predominantly in the ORF1b, which encodes non-structural proteins (nsp). Functional analysis of m6A machinery in regulating PEDV revealed that writers METTL3/14 and readers YTHDF-1 and -2 have an inhibitory role, while FTO has the opposite effect (Chen et al., 2020). Intriguingly, the decoration of the m6A marks in the non-structural regions of the PEDV genome may contribute to innate immune inhibitory.

##### 5.4.4.2 Severe acute respiratory syndrome Coronavirus-2 (SARS-CoV-2)

Regarding the methylome of SARS-CoV-2, the causative agent responsible for the coronavirus Disease 2019 (COVID-19) pandemic, m6A-seq and miCLIP combined technologies have been used to provide single-nucleotide resolution data to show that SARS-CoV-2 bears 8 m6A sites. Moreover, METTL3/14 downregulates virus replication; in contrast to ALKBH5, which upregulates the replication of SARS-CoV-2. Like PEDV, SARS-CoV-2 substantially improves m6A cellular methylome in Vero and Huh7 cells (Liu et al., 2021). Based on the previous data, the m6A epitranscriptomic marks seem to negatively regulate coronaviruses (Liu J. et al., 2024). Another study highlights the influence of prevalent adenosine methylations on the transcriptional activity of SARS-CoV-2 RNA-dependent RNA polymerase (RdRp) (Snyder et al., 2024). Moreover, the authors discussed the impact of nucleoside modification(s) on the pre-steady state kinetics and its functional outcomes. Both m6A and 2′-O-methyladenosine (Am) modifications slow down viral transcription at specific magnitudes, which could affect the maintenance of SARS-CoV-2 transcripts (Snyder et al., 2024).

**FIGURE 2:**
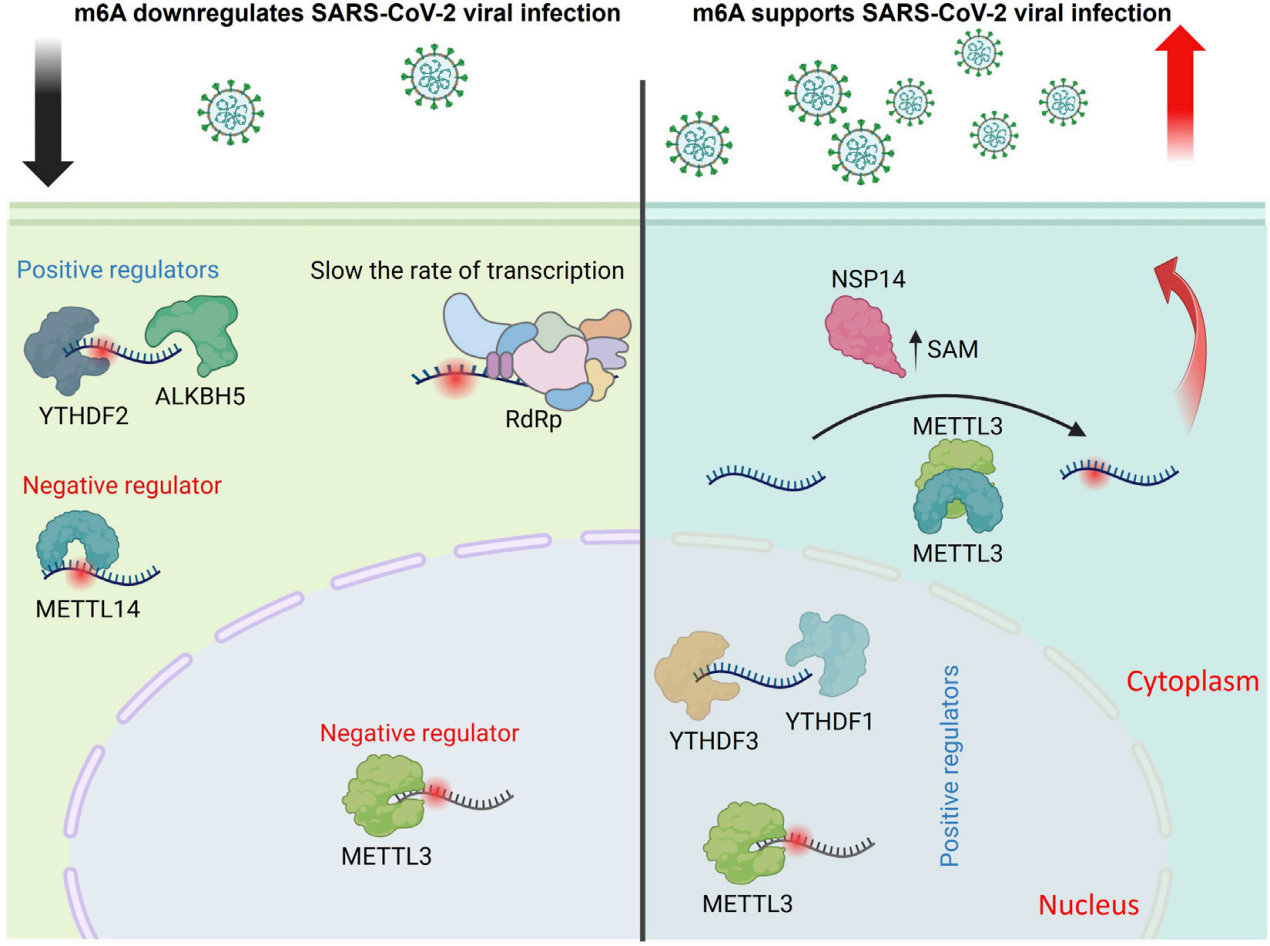
Proposed model illustrating the dual regulatory role of m6A modifications in the SARS-CoV-2 replication cycle. m6A RNA modifications influence multiple stages of SARS-CoV-2 infection, with conflicting findings reported in the literature. Several studies suggest that METTL3 and METTL14 downregulate viral infection, while ALKBH5 and YTHDF2 promote it. m6A marks have been shown to slow viral transcription by impeding the RNA-dependent RNA polymerase (RdRp), indicating a negative regulatory role. Conversely, other reports demonstrate that METTL3, YTHDF1, and YTHDF3 enhance viral replication. Additionally, the viral protein NSP14 promotes S-adenosylmethionine (SAM) expression, increasing m6A-modified mRNAs to facilitate infection, supporting a positive regulatory role for m6A in this context (The figure was created using BioRender).

Notably, another report using SARS-CoV-2 and HCoV-OC43 showed that METTL3 and YTHDF1-3 promote both virus replication in VeroE6 cells and their depletion suppresses viral infection (Burgess et al., 2021). Although the discrepancies are clearly noticed, the difference in the cell line could be the plausible cause, which makes judging the overall impact of m6A in coronavirus regulation challenging. Another report recently argued the positive impact of m6A regulating SARS-CoV-2 infection. SARS-CoV-2 and HCoV-OC43 infections promote host m6A modification by activating the mTORC1 signaling pathway. Machanistically, the viral nsp14 increases the expression of S-adenosylmethionine (SAM) synthase MAT2A in a manner dependent on mTORC1. This mTORC1-MAT2A interaction subsequently enhances the production of SAM, hence boosting m6A methylation of host RNA to facilitate viral replication (Zhou et al., 2025). All these studies uncover the molecular mechanism through which viruses affect m6A methylation of their hosts and explain how viruses utilize host cellular epitranscriptomic modifications to promote their replication (Figure 2).

#### 5.4.5 The role of m6A in regulating viruses belonging to the *Artriviridae* family

##### 5.4.5.1 Porcine reproductive and respiratory syndrome virus (PRRSV)

PRRSV causes reproductive issues and respiratory problems in pigs, leading to significant financial losses for the swine industry. Infection with PRRSV leads to an increase in IL-13 levels within porcine alveolar macrophages. The virus promotes the accumulation of m6A-methylated RNA while simultaneously decreasing the expression of FTO, which in turn enhances IL-13 production, with PRRSV nsp9 playing a crucial role in this regulatory process. Additionally, their findings indicate that the amino acid residues, including D567, Y586, L593, and D595, are vital for nsp9 to stimulate IL-13 production by downregulating FTO expression (Gong et al., 2024). These findings point out the function of PRRSV nsp9 in the FTO-mediated release of IL-13, thereby deepening our understanding of the virus’s effects on the host’s immune and inflammatory responses.

### 5.5 Class V viruses: single-stranded RNA, negative sense viruses

#### 5.5.1 The role of m6A in regulating viruses belonging to the *Pneumoviridae* family

##### 5.5.1.1 Respiratory syncytial virus (RSV)

RSV undergoes m6A modifications, and the major virus structural glycoprotein (G) has been noticed to contain m6A sites. Abrogative silent mutations to these m6A sites enriched on the G gene significantly reduced viral replication kinetics (Xue et al., 2019). Inhibition of the methyltransferase complex decreased gene expression and viral replication, whereas inhibiting the eraser enzymes had the opposite effect. Moreover, YTHDF proteins had a positive regulatory role indicated by enhanced viral gene expression and virion production upon overexpression (Xue et al., 2019). On the other side, others have identified the m6A reader YTHDC1 as a negative regulator of RSV infection. Their findings indicate that YTHDC1 inhibits RSV infection by decreasing the expression of the entry receptor, CX3C motif chemokine receptor 1 (CX3CR1), on the surface of lung epithelial cells (Picavet et al., 2024). These findings could aid in the creation of new therapeutic strategies for managing RSV infection.

##### 5.5.1.2 Human pneumovirus (HMPV)

HMPV, another member *in the Pneumoviridae* family, possesses m6A marks that positively regulate viral replication and gene expression in the same manner and functional relevance indicated in the RSV model (Lu et al., 2020). Interestingly, this model also illustrated that the m6A marks can be exploited to enable viruses to evade innate immune response by escaping recognition by innate immune sensors, including RIG-I (Lu et al., 2020).

#### 5.5.2 The role of m6A in regulating viruses belonging to the *Rhabdoviridae* family

##### 5.5.2.1 Vesicular stomatitis virus (VSV)

The m6A machinery regulates VSV infection by disrupting innate antiviral immunity. Upon VSV infection, the nuclear DEAD-box-46 (DDX46) helicase recruits ALKBH5, which demethylates the m6A marks from key immune modulators. Upon demethylation, mRNAs of these innate immune modulators remain sequestered in the nucleus, inhibit IFN, and promote replication. ALKBH5 knockdown induced IFN production and inhibited VSV replication (Zheng et al., 2017). Others have also reported that ALKBH5 knockdown strongly suppresses VSV replication. Mechanistically, ALKBH5 depletion induces high m6A on α-ketoglutarate dehydrogenase (OGDH) transcripts, negatively affecting their stability. Accordingly, the metabolite itaconate pathway required for viral replication will ultimately be inhibited (Liu et al., 2019). This report highlights the impact of m6A on the cellular metabolome.

An investigation also supported the idea that METTL3 reshapes innate immune responses to accelerate VSV clearance after infection. Overexpressed METTL3 translocates to the cytoplasm, installing extra m6A marks on the VSV RNA. This negatively affects dsRNA formation and dampens the innate immune responses, hence upregulating VSV replication. Upon METTL3 depletion, reduced m6A levels enhance type I IFN expression, ultimately inducing virus clearance (Qiu et al., 2021). In summary, m6A marks play a role in the VSV infection cycle by regulating innate immune responses.

#### 5.5.3 The role of m6A in regulating viruses belonging to the *Phenuiviridae* family

##### 5.5.3.1 Severe Fever with Thrombocytopenia Syndrome virus (SFTSV)

Severe Fever with Thrombocytopenia Syndrome is an emerging infectious disease caused by SFTSV, an infection which is transmitted by tick bites. MeRIP-seq analysis confirmed that SFTSV RNA were m6A modified (Chen Z. et al., 2024; Liu B. et al., 2024). Furthermore, the authors illustrate that YTHDF1 interacts with the m6A modification sites on SFTSV, resulting in decreased stability of SFTSV RNA and reduced translation efficiency of SFTSV proteins. In response, the virulence factor non-structural protein NSs of SFTSV enhances the lactylation, a type of post-translational modification, of YTHDF1, which facilitates its degradation and subsequently promotes SFTSV replication (Liu B. et al., 2024).

#### 5.5.4 The role of m6A in regulating viruses belonging to the *Orthomyxoviridae* family

##### 5.5.4.1 The role of m6A in regulating influenza a viruses (IAVs)

IAVs are nuclear-replicating negative-sense, single-stranded RNA viruses that have been identified to carry m6A marks on their genome since the 1970s. This earlier report indicated, through biochemical RNA labelling analysis that IAV bears 24 m6A sites in the entire segmented genome (Krug et al., 1976). Later, another report indicated that the 24 m6A sites were unequally distributed among the genome of IAV. It has also been identified that the highest m6A marks were on the viral glycoprotein hemagglutinin (HA) and neuraminidase (NA) genes, whereas some genes, such as the polymerase basic 2 (PB2) and nucleoprotein (NP) segments, lack any m6A modifications (Narayan et al., 1987). Nonetheless, due to the lack of m6A topology information, the functional relevance of these marks on the viral RNA remained unclear till recently.

Using photo-assisted crosslinking m6A sequencing (PA-m6A-seq) combined with photoactivatable ribonucleoside-enhanced crosslinking and immunoprecipitation (PAR-CLIP) data revealed that influenza A/Puerto Rico/8/34/Mount Sinai H1N1 (PR8) bears 8/9 m6A sites on viral mRNA/vRNA, respectively (Courtney et al., 2017). They also verified that YTHDF2 and METTL3 significantly enhanced PR8 virus genome replication and gene expression; in this way, the authors suggested the positive regulatory role of m6A in regulating IAV. The potential m6A sites on the HA plus and minus strands were mapped. Using m6A-deficient viruses, the authors revealed that m6A dramatically reduced replication and protein expression in cultured cells and showed reduced pathogenicity *in vivo*, confirming the positive regulatory role of m6A in H1N1 infection.

A recent study revealed that the m6A reader protein YTHDC1 acts as a host factor by interacting with the IAV non-structural 1 (NS1) protein and controlling viral mRNA splicing. By inhibiting NS splicing and reducing nuclear export protein (NEP) expression, YTHDC1 promotes viral replication and increases pathogenicity (Zhu et al., 2023). Our group also reported that through analysis of the conservation patterns of DRACH motifs, which are the canonical motif for deposition of the m6A marks, in viral mRNA m6A sites, indicated that the highest level of conservation was found in H1 sequences, with four DRACHs being preserved across all influenza sequences. In contrast, the conservation and quantity of DRACH motifs are significantly reduced in viral vRNA compared to mRNA. This discrepancy may be attributed to the critical role of m6A modification in the translation and stability of mRNA (Bayoumi and Munir, 2021a).

**FIGURE 3:**
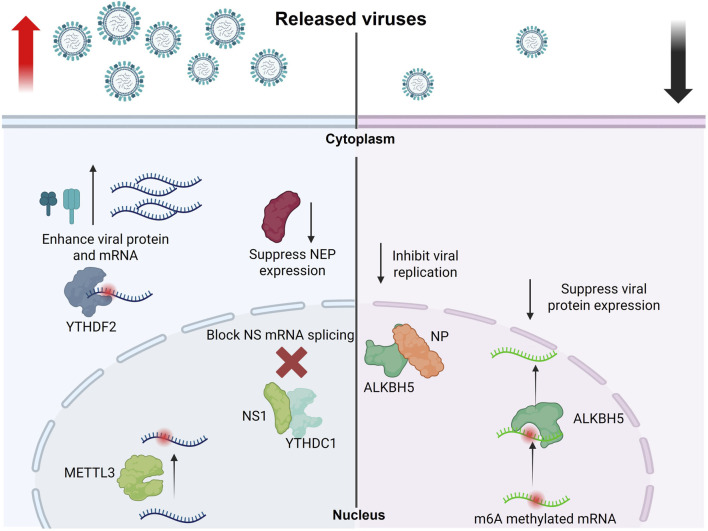
Role of m6A modifications in regulating the influenza A virus replication cycle. This schematic illustrates how m6A RNA modifications influence the replication and gene expression of influenza A virus. METTL3 and YTHDF2 enhance the expression of viral genes and proteins, promoting viral replication. Conversely, YTHDC1 suppresses NEP expression by blocking NS mRNA splicing, which indirectly supports viral replication. In contrast, the demethylase ALKBH5 removes m6A modifications from methylated viral mRNAs and interacts with the viral NP protein to inhibit viral gene expression and replication (The figure was created using BioRender).

Furthermore, our findings indicated that IAV significantly decreased the transcription levels of m6A writers and erasers. In contrast, the regulation of m6A readers was observed to be moderate within the chicken fibroblast cell model. Additionally, we noted structural and genetic differences in the avian m6A machinery compared to humans (Bayoumi et al., 2020). We also reported that the chicken (ch)ALKBH5 inhibits avian IAV replication and viral protein expression. The antiviral function of chALKBH5 relies on its 2OG-(Fe)II-oxy and C-terminal domains. Mechanistically, chALKBH5 directly interacts with the viral NP and, when guided to viral RNA using Cas13b technology (Bayoumi and Munir, 2021b), removes m6A modifications, thereby suppressing viral replication. MeRIP-seq confirmed that H9N2 viral transcripts are m6A-methylated, and recombinant viruses generated using reverse genetics approaches showed that increased m6A levels enhance H9N2 replication (Bayoumi, 2023). In conclusion, all research on IAV highlights the significant role of m6A in avian IAV infections, suggesting that m6A regulators may serve as important antiviral agents (Figure 3).

### 5.6 Class VI viruses: single-stranded RNA containing reverse transcriptase

#### 5.6.1 The role of m6A in regulating viruses belongs to the *Retroviridae* family

##### 5.6.1.1 Human immunodeficiency virus-1 (HIV-1)

All investigated epitranscriptomic studies also confirmed that m6A modifications control the HIV-1 lifecycle in various manners. It has been reported that HIV-1 RNA bears at least 14 m6A peaks in the coding and non-coding untranslated regions (UTRs). Additionally, the host m6A increased substantially upon viral stimulation, and these m6A marks enhanced virus production (Lichinchi et al., 2016a). The mechanistic investigation also indicated that m6A influences gene expression and the nuclear export of viral RNA. Furthermore, METTL3/14 enhanced viral gene expression, while ALKBH5 had the opposite effect (Lichinchi et al., 2016a). Others reported the same conclusion; however, they mapped the m6A marks in the 3′UTR only, and the YTHDFs recruited to viral RNA to promote viral gene expression in CD4^+^ T and HEK-293T cells (Kennedy et al., 2016).

In contrast, it has been shown that YTHDFs inhibited viral production by inhibiting the reverse transcriptase enzyme in primary CD4^+^ T cells (Tirumuru et al., 2016; Lu et al., 2018). A recent study also supports the antiviral role of YTHDF3 in regulating HIV-1 replication in the reverse transcription step. YTHDF3 was incorporated into the released virion capsid protein to inhibit the newly infected cells in this investigation. Accordingly, viral protease degraded the cellular encapsidated protein YTHDF3 to restore optimal infectivity (Jurczyszak et al., 2020).

A recent study examined chemical alterations in HIV-1 RNAs at the full-length, single RNA level, and nucleotide level resolution through direct RNA sequencing techniques. Findings indicate a surprisingly straightforward modification landscape for HIV-1, with three primary m6A modifications identified near the 3′end, which are densely present in spliced viral mRNAs compared to genomic RNAs, and they are essential for sustaining normal levels of HIV-1 RNA splicing and translation. HIV-1 produces a variety of RNA subspecies, each with unique m6A profiles, and the presence of multiple m6A modifications on its RNAs contributes to enhanced stability and resilience in HIV-1 replication (Baek et al., 2024).

The above-mentioned data revealed several discrepancies in the role of m6A in regulating HIV-1 replication. These variations may be attributed to different epitranscriptomic sequencing techniques or cell lines used in individual studies. Moreover, selective and individual investigation of m6A-related enzymes may yield misleading conclusions. Nevertheless, all confirmed that the m6A marks of HIV-1 RNA substantially impact various aspects of the virus life cycle, including latency reversal (Mishra et al., 2024).

##### 5.6.1.2 Murine leukaemia virus (MLV)

Similar to HIV-1, m6A mRNA modifications have been described in the MLV genome, including m6A and m5C. Surprisingly, the authors noticed that these RNA modifications are present at a higher magnitude than those mapped in the cellular counterparts in the given transcripts. Moreover, upon overexpression of YTHDF2, viral replication was enhanced significantly, indicating the proviral role of m6A on MLV infection (Courtney et al., 2019a). These observations further support the notion that RNA modifications like m6A can act as critical regulators of viral replication, potentially offering novel targets for therapeutic intervention in retroviral infections.

##### 5.6.1.3 Human T-cell leukemia virus type 1 (HTLV-1)

HTLV-1 is a retrovirus linked to adult T-cell leukemia/lymphoma (ATLL) and HTLV-1-associated myelopathy/tropical spastic paraparesis (HAM/TSP), which is a progressive neurodegenerative condition. A recent group mapped 3 m6A sites in the 3′ end of the viral genome and specific viral oncogenes, including *tax* and *hbz*. Interestingly, with m6A depletion using writers inhibitor (STM2457), HTLV-1 infection resulted in a reduction of sense-derived viral genes, while simultaneously leading to an elevation in the expression of an antisense-derived *hbz* gene. They also found that YTHDF1 and YTHDC1 m6A readers modulate HTLV-1 tax and hbz activity in different pathways to dictate the fate of viral RNA (King and Panfil, 2025; King et al., 2025).

### 5.7 Class VII viruses: double-stranded DNA containing reverse transcriptase enzyme

#### 5.7.1 The role of m6A in regulating viruses belonging to the *Hepadnaviridae* family

##### 5.7.1.1 Hepatitis B virus (HBV)

Another salient example of the role of m6A in tumour-causing viruses is HBV. The m6A residues have been identified in HBV mRNAs and hepatic tissues collected from HBV patients (Imam et al., 2018). Loss-of-function studies revealed that m6A affects mRNA stability and regulates the pregenomic RNA (pgRNA) and reverse transcriptase (Imam et al., 2018). The m6A-seq analysis also revealed that m6A marks are located within the epsilon stem-loop region. The m6A marks were mapped in both 5′and 3′ends of the pgRNA and the 3′ends of viral transcripts. Using m6A mutational analysis, the authors confirmed that m6A sites located in the 5′stem-loop of the pgRNA regulated efficient reverse transcription, while the m6A sites located in the 3′stem-loop negatively affected the stability of all HBV mRNAs, indicating a dual regulatory role of m6A (Imam et al., 2018).

The same group also confirmed that mutational analysis in the m6A site in the 5′stem-loop of the pgRNA affects RIG-I binding affinity to evade the innate immune system. RIG-I is a crucial member of innate immune sensors that detect mainly viral RNA. Recognizing non-self RNA triggers various proinflammatory cytokines and type I IFN to establish an antiviral response (Kim G. W. et al., 2020; Lu et al., 2020). A summary of the m6A-related protein regulatory role in various viruses is listed in Table 1.

**TABLE 1:** Summary of the roles of m6A machinery in regulating viral infection.

Class	Virus	References	Writers		Readers			Erasers	
			METTL3	METTL14	YTHDF1	YTHDF2	YTHDF3	ALKBH5	FTO
I	HSV-1	Feng et al. (2021), Chen et al. (2024b)	+*	+			+	-	
	HCMV	Winkler et al. (2019)	+			+			
	KSHV	Ye et al. (2017)	+						-
		Hesser et al. (2018)	+/−			+/−			
		Tan et al. (2018)				-			
		Baquero-Perez et al. (2019)	+		+	+	+		-
	EBV	Lang et al. (2019)		+					
		Xia et al. (2021)			-				+
	PRV	Verhamme et al. (2025)							
	Ad5	Price et al. (2020)	+						
	SV40	Tsai et al. (2018)	+			+			
III	RV	Wang et al. (2022)	+						
IV	EV71	Hao et al. (2019)	+	+	+/−	+/−	+/−		-
	CVB1/3	Bonfim et al. (2024), Zhao et al. (2024a), Zhao et al. (2024b)	+	+	+	+	+	-	-
	HCV	Gokhale et al. (2016)	-						+
	CSFV	Chen et al. (2024a)		+		+			
	ZIKV	Lichinchi et al. (2016b)	-		-	-	-		
	CV	Kim et al. (2020a)			-	+	-		
	PEDV	Chen et al. (2020)	-	-	-	-			+
	SARS-COV-2	Liu et al. (2021)	-	-				+	
		Burgess et al. (2021)	+		+	+	+		
	HCoV-OC43	Burgess et al. (2021)	+		+	+	+		
V	RSV	Xue et al. (2019)	+	+	+	+	+	-	-
	HMPV	Lu et al. (2020)	+	+	+	+	+	-	-
	SFTSV	Liu et al. (2024a)			-				
	VSV	Zheng et al. (2017)						+	
		Liu et al. (2019)						+	
		Qiu et al. (2021)	+						
	IAV	Courtney et al. (2017)	+			+			
VI	HIV-1	Lichinchi et al. (2016a)	+	+				-	
		Kennedy et al. (2016)			+	+	+		
		Tirumuru et al. (2016)			-	-	-		
		Lu et al. (2018)			-	-	-		
		Jurczyszak et al. (2020)				-			
	MLV	Courtney et al. (2019a)				+			
	HTLV-1	King et al. (2025)			+/−				
VII	HBV	Imam et al. (2018)	-	-		-	-	+	+

*(+) indicate a positive regulatory effect of the m6A-related protein on the infecting virus model. (−) indicate a negative regulatory effect of the m6A-related protein on the infecting virus model. (+/−) indicates that the impact differs in different cell models. The viruses in each class, according to the Baltimore classification, are indicated.

## 6 Role of epitranscriptomic modifications in regulating the immune response to viral infection

Host innate immunity primarily depends on type I IFN responses to control viral infections. The invading viral RNA is mainly recognized by cellular pathogen recognition receptors (PRRs), including RIG-I (not present in chicken) and melanoma differentiation-associated protein 5 (MDA5); and TLRs-3, -7 and -9. Viral stimulation triggers signals to express IFN-α and -β, which bind to the IFN-α receptor (IFNAR), activating mainly the JAK-STAT signalling pathway. Consequently, it stimulates the transcription of many ISGs to promote competent antiviral responses (Ivashkiv and Donlin, 2014; Santhakumar et al., 2017).

Based on the above-mentioned impacts of epitranscriptomics in regulating various biological and pathological processes, it is not surprising that m6A also directly regulates immune response against viral infections. It has been reported that m6A methylation of viral RNA mediates evasion from RIG-I recognition in various RNA and DNA models. In HPMV, m6A-deficient viruses promote conformational changes in the RIG-I to induce potent immune recognition (Lu et al., 2020). Furthermore, in HBV and HCV, METT3/14 (i.e., writers) depletion decreases m6A levels on viral transcripts, leading to enhanced RIG-I recognition. YTHDFs protein has a protective effect by occupying m6A-containing RNA, hindering RIG-I recognitions (Kim G. W. et al., 2020).

It has also been confirmed that m6A negatively impacts innate immune responses. YTHDF2 and METTL3-depleted cells were associated with enhanced stability of IFNβ mRNA in an m6A-dependent manner. IFNβ mRNA carries m6A sites, which is highly stabilized in low m6A state conditions. Accordingly, normal conditions facilitate virus replications by fast turnover of IFNβ mRNA (Winkler et al., 2019). Similarly, METTL14 depletion leads to enhanced IFNβ mRNA stability and expression, reducing HCMV viral infection. In contrast, ALKBH5 has the opposite effect on viral replication (Rubio et al., 2018).

YTHDF3 also displayed a negative regulatory role in an IFNβ mRNA-independent manner. YTHDF3 promotes the translation of a transcription repressor named forkhead box protein O3 (FOXO3) upon viral infection. That negatively regulates the expression of ISGs. As a consequence, it promoted viral replication, including VSV, encephalomyocarditis virus (EMCV), and HSV-1 (Zhang et al., 2019). In contrast, YTHDF3 (as a model) negatively regulates various viral replication, including HIV-1, HBV, HCV, and ZIKV, as will be fully described later in the study.

All this information significantly indicates the impact of m6A in various biological processes in eukaryotic cells, viral replication, innate immune modulation, and tumour progression/repression (Karandashov et al., 2024). Due to insufficient data availability, a final conclusion about the role of m6A cannot be inferred, especially in the viral lifecycle. Future research may lead to expanding our understanding of the role of m6A regulation of viral infections. This information could also open new avenues to tackle life-threatening viruses not only genetically but also epitranscriptomically.

## 7 Controversies in m6A biology: challenges and future directions

Epigenetic gene regulations are a group of modifications that include histone remodelling, histone tail modifications, and DNA methylation. All these modifications trigger easier accessibility to genes prone to maximal expression than others at a particular moment (Tsai and Cullen, 2020). In contrast, epitranscriptomic gene regulation encompasses chemical modifications added to the RNA. In general, eukaryotic cells exploit the epigenetic and epitranscriptomic chemical modifications on the cellular DNA and RNA, respectively, to control cellular differentiation and normal growth behaviours. Upon dysregulation, the affected tissues are prone to cancer and metabolic disorders (Meyer and Jaffrey, 2017; Roundtree et al., 2017; Huang et al., 2020). Unlike epigenetic modifications, the study of the epitranscriptomic regulatory role in viral infection is still in its infancy. It is difficult to draw conclusions about its accurate impact on virus infection. Intriguingly, m6A mark enhances viral gene expression and replication in certain viruses. In contrast, the same m6A mark has the opposite effect on others (i.e., reduces viral gene expression and virion production), as we indicated earlier. Adding another layer of complexity, m6A marks or m6A-associated proteins can have both pro- and antiviral outcomes in a given virus, as seen for KSHV, HIV-1, and SARS-CoV-2 (Kennedy et al., 2016; Tirumuru et al., 2016; Tan et al., 2017; Hesser et al., 2018; Burgess et al., 2021; Liu et al., 2021). Moreover, cell-type variation effect was also recorded in m6A-related fields, including KSHV, SARS-CoV-2, and HIV-1 (Kennedy et al., 2016; Tirumuru et al., 2016; Hesser et al., 2018).

Additionally, mapping techniques employed for detecting the location of m6A exhibit specific limitations that impede a thorough comprehension of m6A’s function in distinct viral RNA molecules. This situation may lead to an unintentional bias in the sequencing technology and algorithm utilized, raising the question of whether we are observing a complete overview or merely specific components within it (Horner and Thompson, 2024). Adopting alternative sequencing methods could significantly alter our understanding, revealing that certain viruses lack m6A; cytoplasmic viruses serve as a notable example of this phenomenon (Baquero-Pérez et al., 2024). Additional functional analysis is essential to assess the influence of m6A modifications and m6A-related proteins on viral infections and ascertain whether they predominantly aid host defense mechanisms or facilitate viral infection strategies. Addressing this dilemma could provide significant understanding of viral biology and open up therapeutic avenues for zoonotic diseases. Although epitranscriptomic studies revealed discrepancies in their conclusions, all investigations have confirmed that m6A marks impact various aspects of the viral life cycle.

Thus, a call for standardization would be helpful in a way that we can anticipate the role of m6A in biology, cancer, and virology. Adopting the up-to-date sequencing tools for specific viruses would also be beneficial in avoiding discrepancies in the literature. Moreover, adopting more than one functional analysis approach by combining overexpression, knockdown, and knockout technologies to specified m6A-modulators would also help to make a clear picture. Newly identified m6A-machinery inhibitor may help this notion (Bayoumi and Munir, 2021c; 2021b; Hao et al., 2024; Liu X. et al., 2024; Zou et al., 2024).
